# Pattern recognition of microcirculation with super-resolution ultrasound imaging provides markers for early tumor response to anti-angiogenic therapy

**DOI:** 10.7150/thno.89306

**Published:** 2024-01-20

**Authors:** Jingyi Yin, Feihong Dong, Jian An, Tianyu Guo, Heping Cheng, Jiabin Zhang, Jue Zhang

**Affiliations:** 1 Academy for Advanced Interdisciplinary Studies, Peking University, Beijing, China.; 2 College of Future Technology, Peking University, Beijing, China.; 3 State Key Laboratory of Membrane Biology, Peking-Tsinghua Center for Life Sciences, and Institute of Molecular Medicine, Peking University, Beijing, China.; 4 National Biomedical Imaging Center, Peking University, Beijing, China.; 5 Research Unit of Mitochondria in Brain Diseases, Chinese Academy of Medical Sciences, PKU-Nanjing Institute of Translational Medicine, Nanjing, China.; 6 College of Engineering, Peking University, Beijing, China.

**Keywords:** Super-resolution ultrasound imaging, Tumor microvasculature, Tumor heterogeneity, Anti-angiogenic treatment, Pattern recognition

## Abstract

**Rationale:** Cancer treatment outcome is traditionally evaluated by tumor volume change in clinics, while tumor microvascular heterogeneity reflecting tumor response has not been fully explored due to technical limitations.

**Methods:** We introduce a new paradigm in super-resolution ultrasound imaging, termed pattern recognition of microcirculation (PARM), which identifies both hemodynamic and morphological patterns of tumor microcirculation hidden in spatio-temporal space trajectories of microbubbles.

**Results:** PARM demonstrates the ability to distinguish different local blood flow velocities separated by a distance of 24 μm. Compared with traditional vascular parameters, PARM-derived heterogeneity parameters prove to be more sensitive to microvascular changes following anti-angiogenic therapy. Particularly, PARM-identified “sentinel” microvasculature, exhibiting evident structural changes as early as 24 hours after treatment initiation, correlates significantly with subsequent tumor volume changes (|r| > 0.9, P < 0.05). This provides prognostic insight into tumor response much earlier than clinical criteria.

**Conclusions:** The ability of PARM to noninvasively quantify tumor vascular heterogeneity at the microvascular level may shed new light on early-stage assessment of cancer therapy.

## Introduction

Tumor heterogeneity has played a crucial role in therapeutic failures, drug resistance, and even lethal outcomes for cancer therapy 1. The heterogeneity in tumor microvasculature, as a major driver of tumor heterogeneity 2-4, is manifested in irregular microvascular networks and resultant abnormal hemodynamics. These contribute significantly to the selection of more malignant tumor cells with increased metastatic potential and enhanced drug resistance, posing a formidable barrier to cancer therapy in clinics 5.

As a widely used therapy in the clinical management of cancer 6-9, anti-vascular treatment is capable of inducing tumor regression by reducing tumor microvascular heterogeneity through vessel normalization 3, 10, 11. Despite early changes observed in tumor microvasculature, the measurable change in tumor volume often takes months after treatment 12. Therefore, current clinical response criteria defined solely by size can be misleading. As over- or under-treatment would further aggravate tumor invasion and treatment resistance 13, timely evaluation of dynamic changes in tumor vasculature is crucial for effective tumor control, which depends on reliable quantification of microvascular structure and function.

However, several critical technical limitations have been recognized. In clinical practices, histochemistry is invasive and yields no necessary information on vascular function. Meanwhile, the low spatial resolution of clinical imaging modalities 14, such as magnetic resonance imaging (MRI), dynamic contrast-enhanced ultrasonography (DCE-US), computed tomography (CT), and positron emission tomography (PET), makes them futile to resolve microvessels that are more sensitive to anti-vascular therapy 15-17. Thus, effectively evaluating tumor microvascular heterogeneity is challenging.

Recently, super-resolution ultrasound (SRUS) imaging techniques produce vascular and velocity maps noninvasively at the micrometer scale 18-23. However, traditional ultrasound localization microscopy (ULM) relies on very low microbubble (MB) concentrations, long acquisition times, and reliable motion models complying with MB dynamics 18, 19, 21, 23, making it impractical in clinics where high MB concentrations and limited acquisition time are common. Notably, there is growing evidence that chaotic tumor vascularization has undermined the reliability of ULM in evaluating microvascular structure and function 20, 21. That is because the slice thickness of the 2D image plane (elevational resolution) is much larger than the size of microvasculature, leading to significant overlays of complex tumor vessels. Consequently, overlapping MB signals in contrast-enhanced ultrasound (CEUS) images. would result in a more difficult and unreliable assessment of vascular parameters 21.

In recent years, multiple research groups have developed multiple non-localization-based SRUS strategies based on microbubbles with high concentrations, which can substantially enhance the proportion of vascular reconstruction. For instance, the study in 24 estimated higher-order statistical properties of temporal signals at each pixel in CEUS images, which effectively enhances differences in blood-flow dynamics among neighboring blood vessels. Nonetheless, the typical resolution improvement is restricted to 2-fold. To address this challenge, sparsity-based super-resolution ultrasound hemodynamic imaging incorporates the sparsity in the underlying microvasculature and achieves SRUS imaging at a high temporal resolution of 25 Hz 25. Ultrasound diffraction attenuation microscopy proposed by Zhang utilized radiality distribution to preserve microbubble trajectories adaptively 26. Besides, a series of deep-learning based methods were developed to establish mapping relationships between densely distributed MBs (or MB tracks) and localizations 27, 28. However, none of these newly-developed methods could quantify blood flow velocities or flow direction of microbubbles. The microbubble separation method proposed in 29 used multiple bandpass filters to separate MB subpopulations on the basis of their speed and flow direction, permitting robust vascular imaging of high-concentration MBs. Nevertheless, the number of subsets and passband range for each filter were pre-set empirically, and an increasing the number of subsets would dramatically increases the computational cost 29.

In this study, we realized that by introducing the time dimension into the CEUS images and forming a 3D spatio-temporal space (space × space × time), MBs moving in time will appear as continuous tubular structures, defined as trail spread function (TSF). The correlation between the structural characteristics of TSF and MB flow characteristics allows reliable velocity mapping, eliminating the process of localizing or tracking individual MBs, as required in ULM 20, 30. Meanwhile, effective differentiation between complex tumor hemodynamics may be facilitated adaptively based on pattern recognition. Herein, we innovatively proposed a brand-new SRUS paradigm termed pattern recognition of microcirculation (PARM), and attempted not only to achieve reliable capture of 3D microvascular characteristics, but offer a unique perspective on the assessment of tumor microvascular heterogeneity. A 3D programmable ultrasound imaging system with a mechanical scanning device was applied to perform PARM across the entire tumor volume 22, 30. By using the widely accepted vascular maturity index (VMI) and tumor volume as standards, we further sought to evaluate the capability of PARM in tracking 3D vascular dynamics and indicating the progression of tumor during anti-vascular therapy, especially at the early stage.

## Methods and material

### Animal model and anti-angiogenesis therapy

All experimental procedures were reviewed and approved by the local animal care committee of Peking University (AAIS-ZhangJ-8). 30 female BLAB/c nude mice (Beijing Vital River Laboratory Animal Technology; 6-8 weeks old; 15-20 g) were used for inducing the glioblastoma cancer models. The human glioblastoma cell line U87-MG, sensitive to bevacizumab treatment 31, was obtained from the Chinese Academy of Medical Sciences Cell Culture Center (Beijing, China). 1
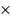
10^7^ U87-MG cells were injected subcutaneously into the right lower hind limb of all nude mice. Tumors reached a volume of ∼100 mm^3^ (mean value) 12 days after tumor cell injection.

The dosing regimen was designed based on previous animal studies 31-33. The clinically used antiangiogenic agent bevacizumab (MedChemExpress, NJ, USA, #HY-P9906) was diluted in phosphate-buffered saline (PBS, pH 7.4, Hyclone, USA), and was administered every other day for 12 days (Figure S3) via intraperitoneal injection at a dose of 10 mg/kg for animals in the treatment group. Meanwhile, mice in the control group received an equivalent volume of PBS.

### Treatment plan and assessment of tumor response

21 mice with approximately tumor diameter of 6 mm (mean volume ∼100 mm^3^) at day 12 (baseline) were selected and randomly allocated to two groups (Figures S2 and S3).

The longitudinal assessment of the anti-angiogenesis therapy group (15 mice) was designed for monitoring treatment progression with histology assessment. 3 mice were killed at baseline and tumors were excised for ex vivo analysis. On days 14 and 18, 3 mice from every group were excised for histological staining. In particular, to correlate in vivo ultrasound imaging results with ex vivo analysis, 3 mice from the control group underwent ultrasound imaging before being killed for histological analysis on day 14. For the longitudinal intraindividual ultrasound imaging group (6 mice), ultrasound imaging was performed on days 12, 14, 18, and 24. All 6 mice were killed and tumors were excised for histological staining after ultrasound imaging on day 24, to correlate in vivo ultrasound imaging results with ex vivo analysis.

Tumor diameter and mice weight were measured every 2 days with a calliper and an electronic scale. Tumor volume was calculated as (1) where 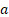 and 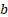 are the long and the short axes, respectively.

*V* = *ab*^2^/2 (1)

Tumor response was evaluated by measuring the relative (to baseline) change in tumor volume following treatment 34-38, which was calculated as (8), where 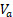 and 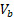 are tumor volumes at baseline (day 12) and 12 days after the treatment initiation (day 24), respectively, which is similar to response evaluation criteria in solid tumors (RECIST) guidelines used in current clinical practice 39.

Δ*V* = (*V_b_*-*V_a_*)/*V_a_* (2)

### Ultrasound imaging protocol

Mice were initially anesthetized in an induction chamber with 3% isoflurane for 1 minute. Subsequently, they were transferred to a heated imaging platform, and inhalable anesthesia (1% isoflurane) was continuously delivered via nose cones throughout the ultrasound imaging procedure. To ensure stable acquisition, all four limbs of the mice were securely affixed to the imaging platform, with shock-absorbing cotton positioned beneath the tumor.

A Verasonics system (Vantage 256, Verasonics Inc) was synchronized with a mechanical scanning system to translate a linear array transducer (L22-8, Kolo Medical Inc) along the elevation direction, with a step size of 200 µm. A center frequency of 15 MHz and a transmit voltage of 15 volts were used for plane-wave ultrasound transmission. Imaging was performed using a 9-angle plane-wave compounding (-12° to 12°, 3° increment) at a frame rate of 500 Hz.

Self-made MBs comprised of octafluoropropane (OFP) gaseous core and lipid shell were reconstituted with a solution at approximately 1.8 × 10^9^ MBs per ml (Details provided in Supplementary Materials). The microbubbles were diluted as 2 × 10^8^ MBs per ml and 100 μL solution was injected through the tail vein.

Imaging acquisitions were acquired at the MB concentration plateau following the bolus peak. For each ultrasound image plane, 1,500 images were acquired, and 20~30 planes were sampled throughout each tumor mass. Acquisitions were stored and the spatiotemporal-based singular value decomposition (SVD) filter was applied to the In-phase and quadrature (IQ) dataset to extract the flowing MB signal 40, thereby generating the CEUS image sequence 41. To eliminate motion artifacts, frames displaying significant movement were excluded before the SVD filtering, assessed by cross-correlation between B-mode frames. Since the subcutaneous tumor models were implanted on the hind limbs, where motion artifacts due to respiration are minimal, we excluded less than 0.1% of the data in each section. Frames before and after frame intervals, resulting from data exclusion, were processed separately to avoid velocity estimation errors.

The tumor border was outlined manually on B-mode images and subsequent processing steps were performed within the segmented area.

### Offline processing of SRUS

The PARM strategy generally divides flowing microbubbles in CEUS images into multiple sparser subsets, by performing adaptive K-means clustering in the functional feature space composed of tumor hemodynamic features. Then, multiple super-resolved microvascular subgroups with different flow characteristics are reconstructed through separate SRUS imaging of microbubble subgroups (Supplementary Methods). It is important to note that PARM can be seamlessly integrated with many existing SRUS method, not limited to ULM. PARM does not require tracking implementation, as it can quantify the instantaneous microbubble velocity and direction through TSF.

After identifying all patterns, the MB subgroup with the highest speed was extracted. Then, the corresponding microvascular subtype, named sentinel microvasculature, was obtained by super-resolution radial fluctuations (SRRF), which was carried out with the nanoJ plugin for ImageJ 42. Parameters including 'ring radius', 'radiality magnification', and 'axes in the ring' were set as 8, 3, and 8 respectively. ULM maps were reconstructed through the ThunderSTORM plugin as previously established 24, 25 (Details in Supplementary Methods). The super-resolved vasculature reconstructed from all the rest MB subgroups were defined as non-sentinel microvasculature.

### Assessment of imaging parameters

As shown in Figure S5, assume that there are 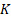 identified patterns in the functional feature space, the PARM-derived heterogeneity parameters include pattern count (PC), pattern distance (PD), and pattern variance (PV).

The number of identified patterns is defined as the PC. The maximum value of the distance between every two clustering centers is defined as the PD:

max (*d_1,2_*, *d_1,3_*, …, *d*_N-1_*,*_N_) *N* = *K*(*K*-1)/2 (3)

*d*_N-1_*,*_N_ represents the distance between the centers of cluster N-1 (C_N-1_) and cluster N (C_N_) in the functional feature space.

For every pattern *n* (*n* = 1, 2, 3, …, *K*), the ratio of the number of feature points *M_n_* to the number of all feature points in the functional feature space is calculated as:

*R_n_* = *M_n_* ⁄ (*M*_1_ + *M*_2_, …, + *M_K_*) (4)

Then, the variance of *R*_1_, *R*_2_, …, and *R_K_* is defined as PV. Specifically, PC reflects the diversity of blood flow dynamics. PD represents the range of spatio-temporal fluctuations in flow dynamics, while PV indicates both the abundance of hemodynamics and the balance of blood flow distribution among different patterns.

For comparison, traditional vascular parameters, including vessel density (VD), vessel number (VN), node number (NN), vessel number per pixel (VNP), vessel tortuosity (VT), mean speed (MS), speed entropy (SE), and orientation variance (OV) were calculated (Supplementary Methods).

The relative (to baseline) change of each indicator at every imaging time point (days 14, 18, and 24) was expressed using the following formula 32:

Relative change = (value at day *N* - value at day 12) / value at day 12 (5)

All morphological, functional, and heterogeneity features of microvessels were calculated via MATLAB 2020b.

### Statistical Analysis

For all bar graphs shown, data are expressed as mean ± standard error of the mean (SEM, n = 3 per group). Sample sizes were chosen according to our experience from previous studies and considering the ethical demands to keep the animal number as low as possible. All analyses were performed using GraphPad Prism 5.0 (GraphPad Software, San Diego, CA, USA) and MATLAB 2020b (MATLAB, MathWorks, Natick, MA, USA).

Shapiro-Wilks goodness-of-fit hypothesis at the level of P = 0.05 were performed on all parameter distributions to check for data normality.

Statistical significance among three tumors groups (n = 9 in total) was evaluated using either the Kruskal-Wallis One-Way ANOVA test or the One-Way ANOVA test. Subsequently, Dunn's post-test or Tukey's honestly significant difference test was employed accordingly to correct for multiple comparisons. Spearmen's correlation coefficients were calculated between the histology-derived parameters and vascular metrics (n=9).

In the longitudinal study, differences in vascular parameters during anti-angiogenic treatment were assessed through two-way ANOVA with Bonferroni correction.

To investigate whether early morphological changes of PARM-identified sentinel microvessels could predict later treatment response, we examined the correlation between relative (to baseline) changes of VT, VN, VNP, NN, and VD calculated from sentinel microvessel on day 14 with VMI / tumor volume changes (ΔV) on day 24 31. Unless otherwise noted, correlations were calculated using a linear least squares regression and reported as Spearmen's correlation coefficient. A Bonferroni-corrected significant difference test was applied to account for multiple comparisons.

In all cases, statistically significant differences are denoted with asterisks (* = P < 0.05, ** = P < 0.01, *** = P < 0.001).

### Immunohistochemistry

After the mice were killed, we sampled multiple (> 3) longitudinal section from the central region of every tumor and performed pathological staining. Prior to analysis, the images were reviewed, and slides displaying evident non-specific staining or tissue folding were excluded. Subsequently, one histopathological section from the tumor center was subjected to analysis.

Immunostaining of endothelial cells in tumor sections was performed with a rat anti-mouse CD31 (Servicebio, GB113151) for vessel density analysis. Smooth muscle cells and pericytes were labeled using biotinylated anti-α-smooth muscle actin (αSMA, Servicebio, GB13044) for vessel maturation analysis. Nuclei were counterstained with 4,6-diamidino-2-phenylindole (DAPI). Fluorescent micrographs were obtained with a Nikon Eclipse C1 microscope and DS-U3 camera control unit (Nikon, Tokyo, Japan).

Tumor vessel density (VD-H) was calculated by dividing the area of CD31-positive structures by the area of the manually selected tumor region 21, 43-48. We then calculated the ratio of CD31 and αSMA positive regions to only CD31-positive regions to determine the VMI (VMI-H) 46, 49, 50.

All analyses were performed with MATLAB 2020b.

## Results

### PARM distinguishes morphological and functional patterns of tumor microvasculature

**Figure 1:**
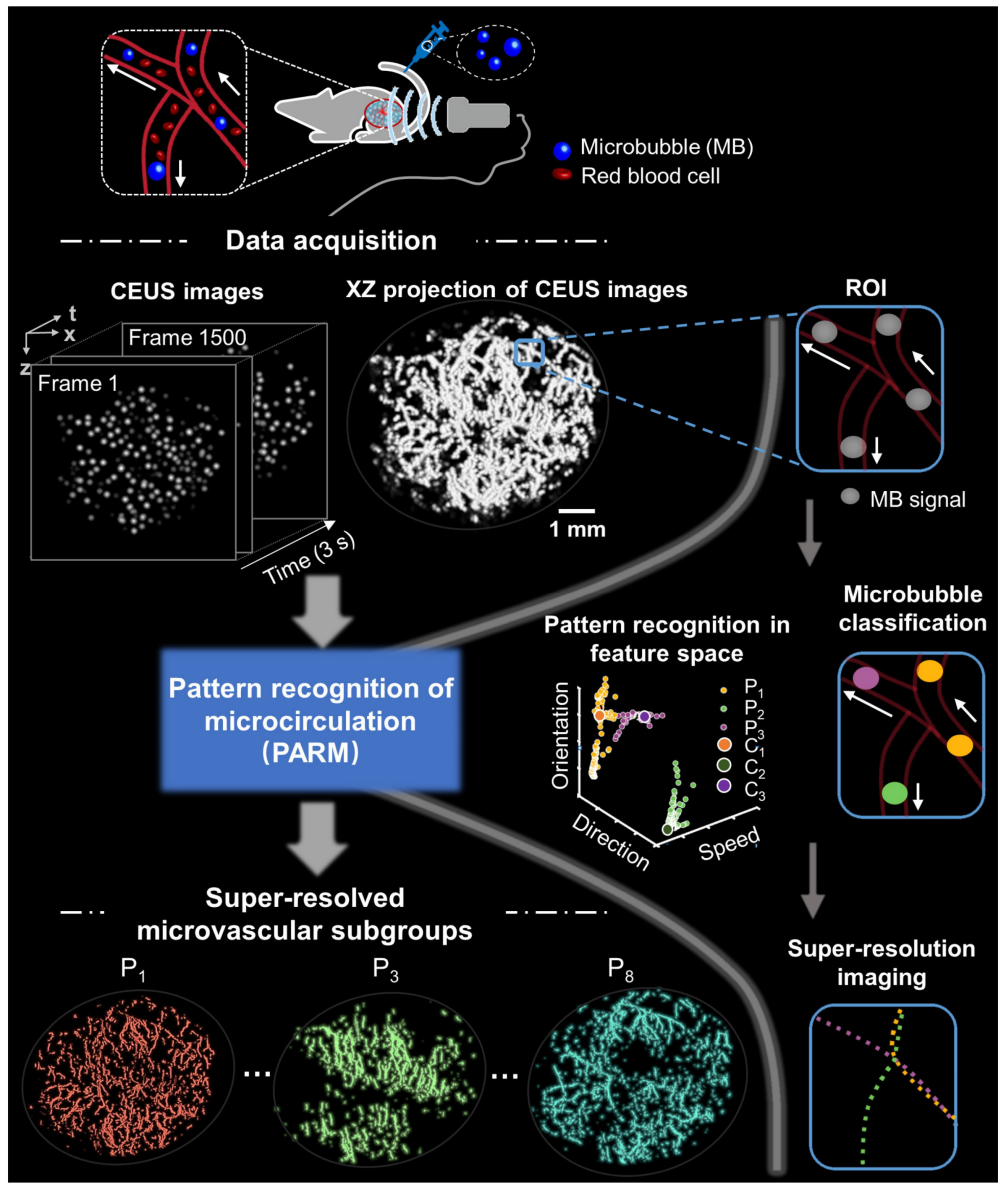
** Schematic diagram of pattern recognition of microcirculation (PARM) process.** Spatio-temporal alignment of successive 2D contrast-enhanced ultrasound (CEUS) images is conducted to form a 3D spatio-temporal matrix. Then by performing clustering analysis in the functional feature space composed of flow characteristics of MBs, PARM achieves pattern recognition of tumor hemodynamics. Correspondingly, microbubbles are classified into multiple subsets and super-resolved microvascular subgroups are reconstructed separately. Clustering centers are labeled as C_1_, C_2_, and C_3_. Pixel-level functional features in the functional feature space are colored accordingly as P_1_, P_2_, and P_3_.

Figure 1 illustrates the workflow diagram of PARM. Unlike ULM, which tracks individual MBs to map blood flow velocity, PARM can directly measure the instantaneous flow characteristics of MBs through their trajectories in 3D spatio-temporal space. Meanwhile, PARM can categorize chaotic tumor microcirculation by hemodynamics and morphology, thus generating multiple super-resolved vascular maps (Figure 1, Supplementary Video 1).

**Figure 2:**
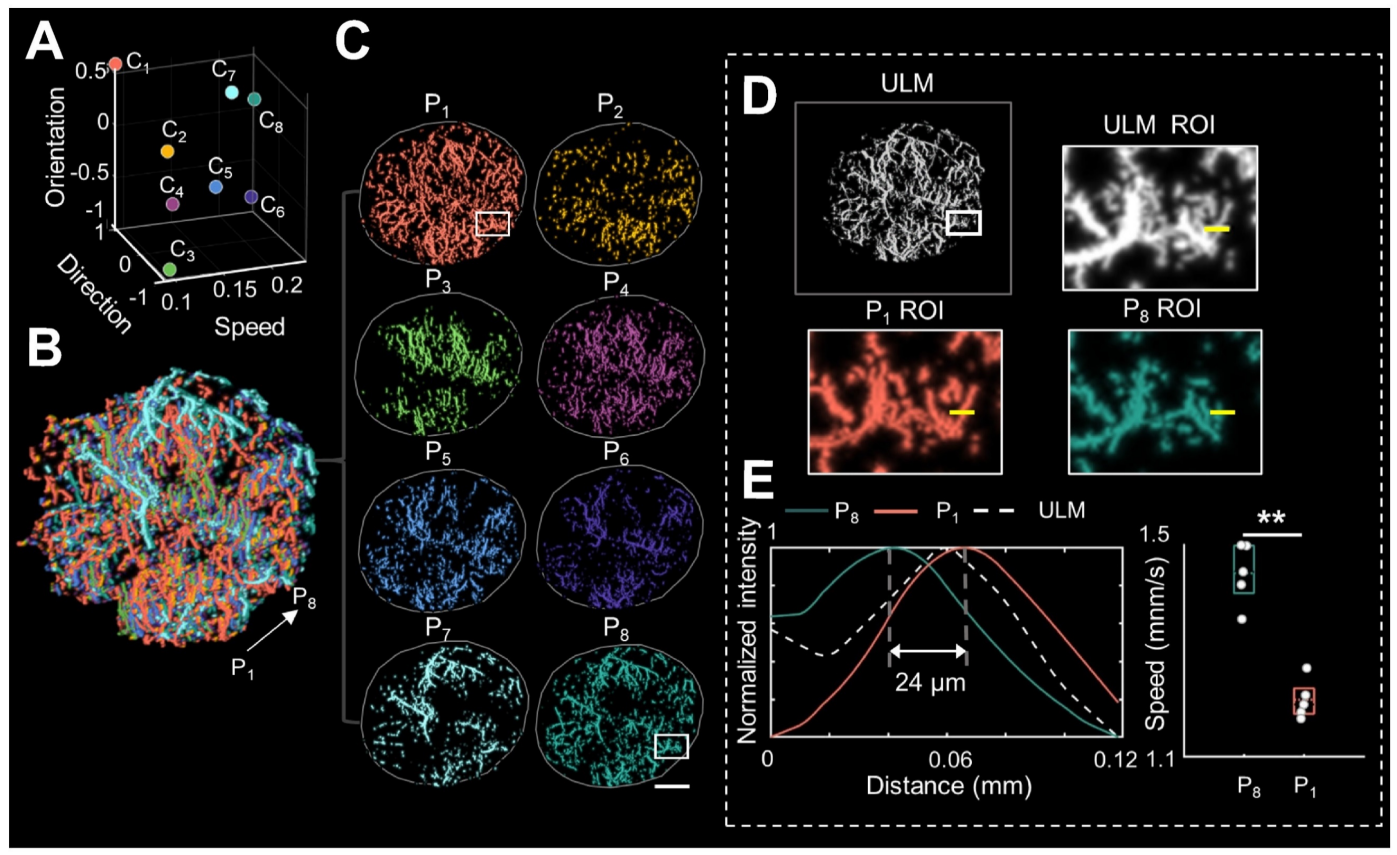
** Pattern recognition of microcirculation (PARM) can clearly distinguish complex tumor microvessels unresolved by ultrasound localization microscopy (ULM).** (A) Eight cluster centers are demonstrated in the feature space. C_1_ represents the clustering center of pattern number 1. The speed values were normalized. (B, C) Correspondingly, multiple MB subsets revealing different hemodynamic features are localized separately, generating eight color-coded super-resolved vascular maps. Tumor boundaries are outlined as gray circles in each color-coded vascular image (C). P_1_ stands for pattern number 1. (D) Magnified regions of interest (ROI, white rectangles) from PARM-identified microvascular subgroups and ULM-reconstructed vascular map. (E) The intensity profiles and microbubble speed distribution of patterns 1 and 8 from manually selected line segments (D, yellow lines). Colored MB subsets are shown in Supplementary Video 1. White color bar: 1 mm.

Notably, distinguished blood flow patterns enable differentiation of non-connected vessels overlapping at the same location in the 2D XZ image plane (Figure S1). This guarantees a reliable quantification of flow characteristics under high MB concentrations (Figure S4), which can be commonly induced by dense tumor vasculature and high imaging slice thickness 25, 29. For in vivo analysis, PARM is capable of distinguishing trajectories of microbubbles with different local blood flow velocities (Figure 2E, P < 0.05) separated by a distance of 24 μm (Figure 2E). In both simulated and in vivo data, ULM fails to provide accurate morphological and functional details of the microvasculature due to unreliable localization and tracking of highly overlapping MBs (Figure 2E, Figure S4).

Moreover, changes in tumor hemodynamics and morphology can be directly reflected in the pattern distribution in PARM-reconstructed functional feature space (Figure S5), quantitatively evaluated by PARM-derived heterogeneity petameters (Figure S5A). Compared to the isotropic flow, distinct flow directions result in a 59% increase in PD (B and C, from 1.55 to 2. 46). Meanwhile, an additional streamline with different flow dynamics increases both PC (B and D, from 2 to 3) and PV (from 0.40 to 0.55). Furthermore, uneven distribution of blood flow, i.e., unbalanced functional pattern distribution, causes a 65% rise in PV (D and E, from 0.55 to 0.91). These indices offer unique perspectives for revealing the complex spatiotemporal heterogeneity of tumor hemodynamics, which has not been assessed by any other noninvasive imaging technique.

### Histological assessment correlates with PARM-derived heterogeneity metrics

**Figure 3:**
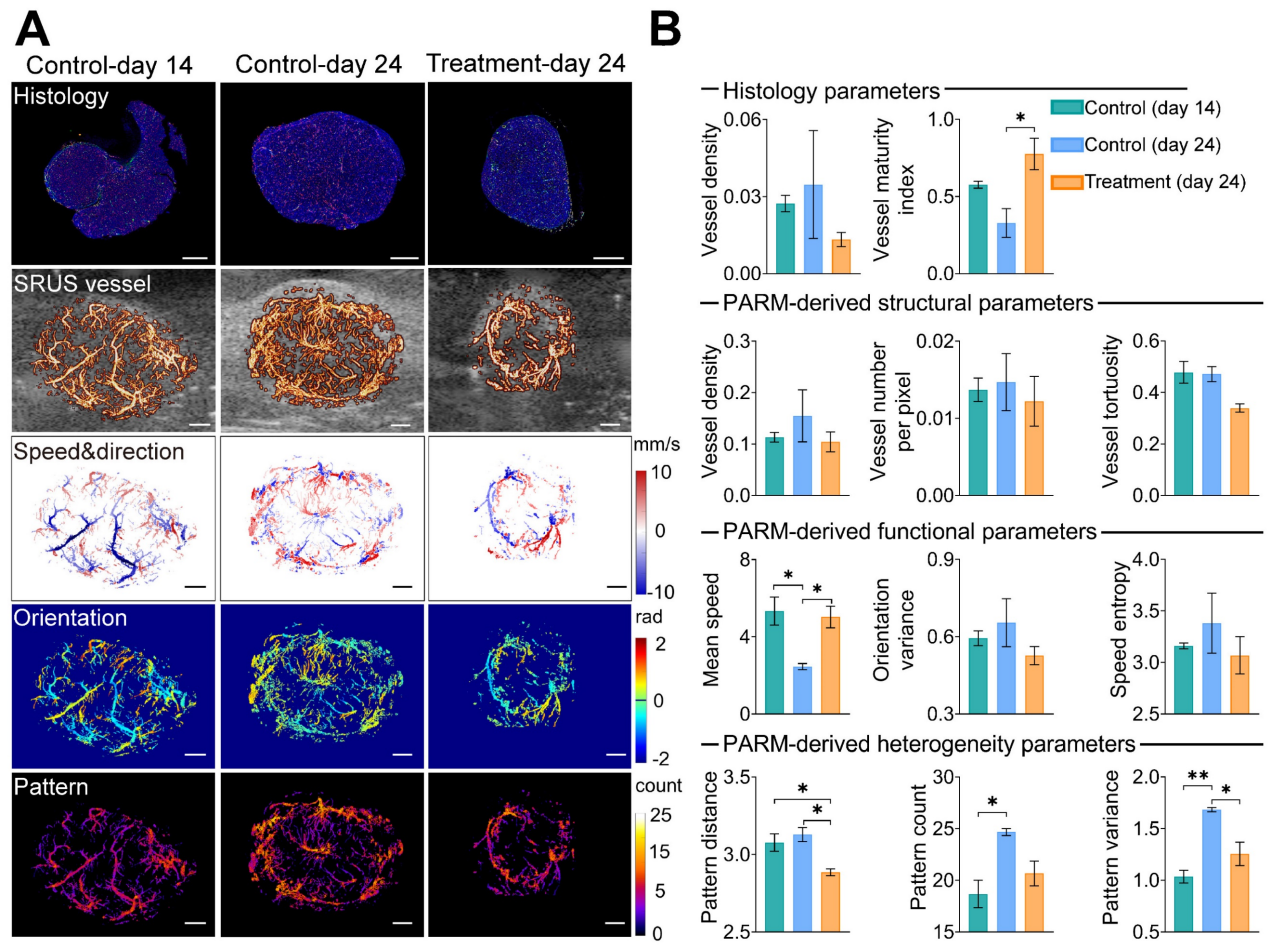
** Pattern recognition of microcirculation (PARM)-derived heterogeneity parameters compared with traditional vascular parameters and histological assessment.** (A) shows CD31(red), αSMA (green), and DAPI (blue) staining results of tumor center slices from the typical control tumor on day 14, control tumor on day 24, and treatment tumor on day 24. The color-coded parameter maps from the corresponding imaging sections represent super-resolved vascular architecture (SRUS vessel, red) overlaid on the B-mode images (gray), XZ plane maximum projection of speed and axial flow direction (speed & direction), lateral flowing angles (orientation), and the number of patterns (pattern). (B) Histological-derived vascular parameters, PARM-derived heterogeneity, functional, and structural parameters were compared across three groups. Statistical significance among three tumor groups was tested using Kruskal-Wallis One-Way ANOVA test or One-Way ANOVA test, with Dunn's post-test or Tukey correction (* = P < 0.05, ** = P < 0.01). Scale bar: 1 mm.

To assess the reliability of the PARM-derived heterogeneity parameters in differentiating tumors with distinct physiological states (3 control tumors at day 14, 3 control tumors at day 24, and 3 treatment tumors at day 24, Figures S2 and S3), we compared them to vessel density (VD-H) and vessel maturity index (VMI-H) measured from gold-standard fluorescent histology quantifications (Figure 3). Bevacizumab significantly improved vessel maturation in the treatment tumors, with increased coverage of αSMA-positive cells (P < 0.05), and a smaller area of CD31 staining, compared with the control tumors. Consistent with these findings, lower VD and VT were observed in the super-resolved microvasculature of typical bevacizumab-treated tumors, while higher SE and OV were demonstrated in control tumors at day 24 (Figure 3B), indicating inhibited formation of new tumor vessels with anti-angiogenic therapy 33.

However, except for MS metric, most of the above structural and functional indices show evident variances among individuals due to the well-known heterogeneity of glioblastoma 51. Even pathological quantified VD fails to distinguish these tumors. In contrast, PV performs the best in discriminating three divergent statuses of tumors. Lower PV in the treatment tumors versus the control coincidentally supports previous findings of anti-vascular drugs in reducing tumor heterogeneity 11. Meanwhile, there is a high correlation between VMI-H and PARM-derived heterogeneity metrics (Table 1, PD, r = -0.77; PV, r = -0.67), implying tumor vascular heterogeneity features are strongly related to vascular maturation. In contrast, ULM-quantified functional and structural parameters show no significant differences among these three groups (Figure S6), and fail to exhibit high correlations with VMI (Table 2). No strong correlations were found between histology-derived vessel density and PARM-derived heterogeneity parameters.

The above evidence suggests that the PARM-derived parameters can effectively reveal differences in vascular heterogeneity among tumors, enabling accurate assessment of tumor vascular normalization.

### Monitoring anti-angiogenic therapy using PARM

**Figure 4:**
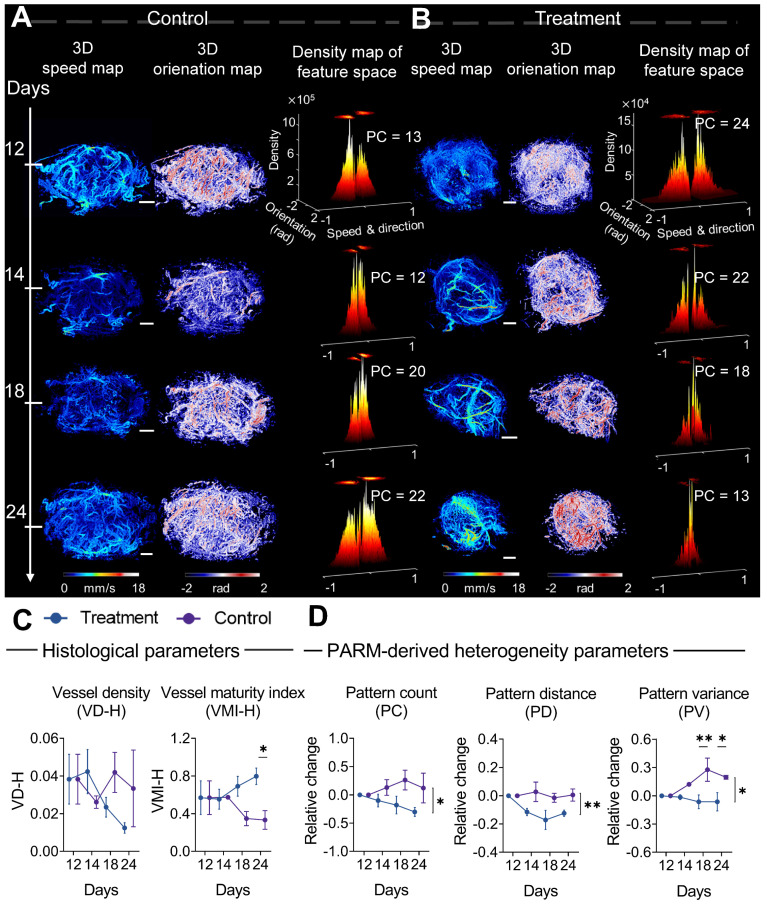
** Progressive response of tumor microvascular heterogeneity to anti-angiogenic therapy.** (A-B) Representative three-dimensional (3D) morphological and functional maps of tumor microvasculature from typical bevacizumab (B) and PBS-treated tumors (A) during treatment. The 3D microvasculature is shown as a 2D maximum-intensity projection. Corresponding density distribution in the pattern recognition of microcirculation (PARM)-reconstructed functional feature space is demonstrated accordingly, with 2D projection shown above the 3D density plots. The speed values were normalized for demonstration. (C, D) The longitudinal changes of PARM-derived heterogeneity parameters and pathological indicators. Differences in parameters during treatment were determined by two-way ANOVA with Bonferroni correction (* = P < 0.05, ** = P < 0.01). White scale bar: 1 mm.

From a pathological perspective, the difference in the trend of vascular maturation between two groups gradually became evident in the mid to late stages of treatment (Figure 4C). Until the 12th day of treatment, a significantly higher VMI was observed in the treatment tumors compared to control tumors (Figure S2B, Figure 4C). These results are consistent with previous findings that bevacizumab has the capability of improving pericyte coverage while decreasing tumor heterogeneity 11, 52. Coincidentally, in the PARM-reconstructed functional feature space, blood flow heterogeneity is suppressed, showing decreased PC in the treatment group (Figure 4B), whereas the control tumors maintain a high level of heterogeneity (Figure 4A).

The dynamic changes in PARM-derived heterogeneity parameters further substantiate the above results. It can be distinctly observed that during the 12-day treatment period, the trends of all heterogeneity parameters in mice treated with bevacizumab significantly differ from those in the control group. For the control tumors, the heterogeneity parameters increased compared to the baseline, while they exhibited a declining trend in the treated tumors (P < 0.05). Notably, the relative (to baseline) changes in PV are significantly different between the two groups at days 18 (P < 0.01) and 24 (P < 0.05). It is worth noting that, while the treatment group displayed an overall declining trend in histology-quantified vascular density, the substantial variance among control tumors resulted in no statistically significant difference between two groups (Figure 4C), highlighting the interference of tumor heterogeneity on conventional quantitative metrics.

Compared to changes in vascular features, no statistically significant difference appeared in the tumor volume change between two groups until day 20 (Figure S3), underscoring the delayed demonstration of the anti-vascular effect quantified by traditional clinical criteria 12, 35, 36, 52, 53. Those results have demonstrated the feasibility of the PARM-derived heterogeneity parameters in sensitively monitoring tumor progression during anti-vascular treatment.

### “Sentinel” microvasculature indicates early treatment response during bevacizumab treatment

Considering the critical role of blood supply in tumor development, we selectively isolated microbubble signals with the highest blood flow velocity from all PARM-identified functional patterns. The corresponding reconstructed microvascular map was referred to as “sentinel” microvasculature. Further quantitative vascular structure analysis of sentinel microvasculature was performed to observe the therapeutic effects of anti-angiogenic drug on critical supply vessels.

**Figure 5:**
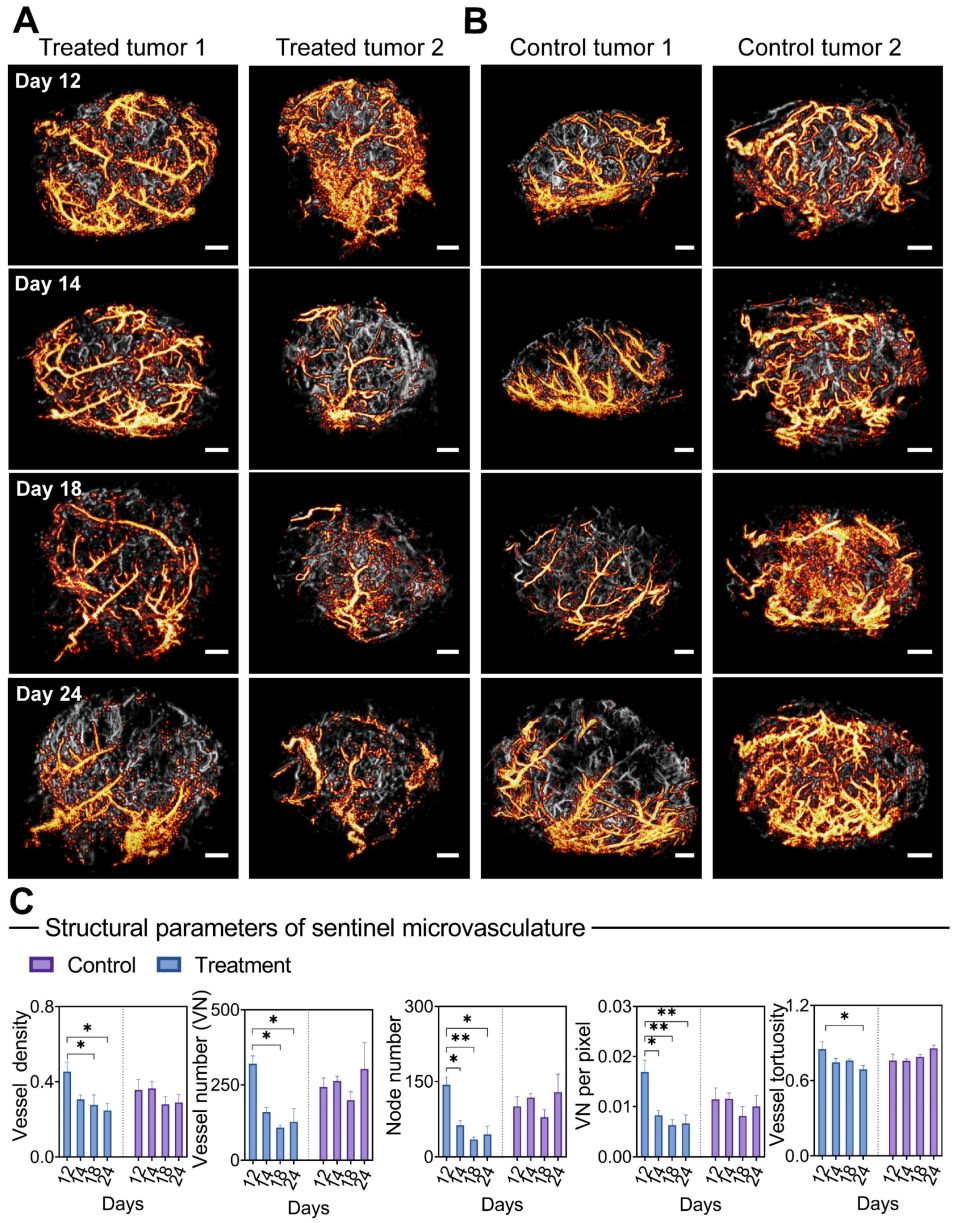
** Morphological changes of sentinel microvasculature during anti-vascular treatment.** (A-B) Representative 3D sentinel microvasculature (orange) in treatment (A) and control (B) tumors are shown as a 2D maximum-intensity projection and superimposed on the microvasculature of the overall tumor (gray). (C) Morphological features of sentinel microvasculature during treatment. Differences in vascular parameters were determined by two-way ANOVA with Bonferroni correction (* = P < 0.05, ** = P < 0.01). Scale bar: 1 mm.

Surprisingly, only 1 day after the first injection, bevacizumab induced a profound vascular normalization effect in the super-resolved sentinel microvasculature (Figure 5A), manifested by substantial reductions in the NN and VNP (P < 0.05, Figure 5C). Significant decreases in VN, VD (days 18 and 24, P < 0.05), and VT (days 24, P < 0.05) were observed subsequently. In comparison, reversed trends in VN, NN, and VT are observed in the sentinel vessels of control tumors. In comparison, the morphological changes of non-sentinel vasculature were not statistically significant (Figure S7). This highlights the unique susceptibility of sentinel microvasculature to anti-angiogenic therapy.

Moreover, we carried out a correlation analysis of early (24 hours after the first dose) structural changes in sentinel microvessel, together with ΔV (relative change of tumor volume compared to baseline) and VMI at day 24 (Table 3). Remarkably, high positive correlations were found between the morphological changes of sentinel microvasculature and ΔV, including VT (r = 0.86), VN (r = 0.94*), VNP (r = 0.87), and NN (r = 0.92*), accompanied by negative correlations with VMI. In particular, no strong correlation was found between the changes of whole tumor vascular morphology and ΔV or VMI.

These interesting findings indicate the feasibility of sentinel microvasculature in noninvasive prognostic of antiangiogenic therapy effects within merely 24 hours after the first treatment.

## Discussion

The heterogeneity of tumor vasculature has not been fully interpreted or explored by traditional clinical imaging modalities so far, due to the unresolved morphology and hemodynamics of tumor microcirculation. Additionally, a lack of quantitative measurements on the tumor microvascular heterogeneity is a concern. SRUS imaging techniques offer new non-invasive approaches for visualizing microvascular structure and function 18, 20-23. However, their reliability has been compromised by chaotic tumor vascularization, particularly for clinical applications 21, 29.

To address this issue, the proposed PARM employs unsupervised K-means clustering in a functional feature space constructed with blood flow characteristics. This enables the easy distinction of characteristically chaotic tumor vasculature based on their functional behaviors (Figure 1). Consequently, this approach effectively offers a possibility to decompose overlapping microbubbles from diverse microflows, which ensures a more reliable mapping of tumor hemodynamics and vascular structures (Figure 2, Figures S1 and S4).

Meanwhile, by quantifying pattern distribution in the functional feature space, PARM offers a brand-new perspective on assessing tumor microvascular heterogeneity. As tumor consists of a society of quasi-stable cellular populations 54, whose status are directly determined by the local blood supply 55, it is possible to track the physiological state of the tumor through PARM. Moreover, novel heterogeneity parameters derived from PARM demonstrated high sensitivity in differentiating among tumors in various physiological states (Figures 3 and 4), whereas traditional vascular metrics proved inadequate due to the intricate nature of blood supply within tumors. These observations were consistent with previous findings of bevacizumab's effect in normalizing blood flow 56, and an elevated degree of internal heterogeneity during tumor growth 57, 58. Meanwhile, compared to PC and PD, PV demonstrated the highest sensitivity in monitoring microvascular heterogeneity changes (Figures 3 and 4), for PV assesses both the variety of functional patterns and blood flow distributions, thus more effectively reflecting bevacizumab's effect on the homogeneous remodeling of disorganized tumor blood flow. Moreover, our experimental results also demonstrated different PC values in baseline tumors (Figure 4), indicating significant intertumoral heterogeneity 59.

In particular, PARM endows a capability to identify key vascular subgroups (sentinel microvasculature) within densely packed capillaries, exhibiting high sensitivity to anti-angiogenic agents. The early changes in morphological metrics of sentinel microvessels displayed a strong correlation with the subsequent tumor response at day 24 (Table 3). Therefore, these findings may prospectively contribute to timely clinical decision-making, providing valuable insights into therapeutic effects. In this proof-of concept study, PARM captured dynamic changes of sentinel microvasculature over long treatment process, despite noticeable variations and changes in tumor size and deformation. Compared to previous strategies premised on voxel matching 60, 61, this may suggest potential clinical benefits in longitudinal monitoring of treatment response 61. Previous studies have indicated that hyperpermeable mother vessels are the dominant subpopulations of tumor vessels susceptible to anti-angiogenic therapy in heterogeneous tumors, which show rapid remodeling and capillary pruning following bevacizumab injection 16, 53, 62, and subsequently trigger tumor regression. These findings align with our observations in the sentinel microvessels. Nevertheless, additional evidence is required for further confirmation.

To ensure reliable clustering analysis, MB distortion resulting from overlapping microbubbles was eliminated during PARM analysis (Supplementary Methods). Frames with out-of-plane movements were excluded to prevent motion-induced errors. The categorized MB subgroups can be pre-reviewed before further processing to empirically eliminate further interferences. Due to a lack of pulsatile blood flow induced by vascular compression and collapse within tumors 63, we assume that the impact of the blood pulsation in the tumor would not likely to affect the feasibility of microbubble separation, as preliminary validated in 29.

As a common challenge, it is difficult to determine whether the diameter and velocimetry of the reconstructed vessels are accurate, as no other in vivo vascular imaging modality can provide a golden standard at a similar resolution 20. Although the use of high-concentration MBs in this study maximizes the portion of the vessels included in the scan within a given acquisition time, PARM cannot ensure a full coverage of the vasculature 25, which is a widely-recognized limitation for CEUS imaging 20, 25. For histological analysis, distortion caused by histology preparation could affect the choice of matching location for comparison 64. Significant variations in histology-derived vascular density are depicted in Figures 3 and 4, particularly in control tumors at 24 days, which may account for its weak correlations with parameters derived from PARM and unreliable assessment of anti-angiogenic drug treatments.

As the effectiveness of PARM relies on the differences in two-dimensional projected velocity/flow direction, the results are influenced by the current distribution of measured flow dynamics, which is fundamentally affected by the imaging angle in 2D imaging. The challenges encountered in 2D ultrasound imaging, including overlapping MB signals caused by low elevational resolution, out-of-plane motion, and velocity measurement, can be circumvented through effective 3D ultrasound imaging 20. However, the implementation of matrix arrays poses technical challenges and is costly 20, 65. Considering the previous successful application of the clinically introduced linear motion stage 22, 30, 66, 67, the combination of PARM and the stepwise motorized motion stage in this study may provide an optimal solution in current clinical scenarios, enabling rapid and reliable assessment of 3D tumor microvessels within 1 minute acquisition time. Moreover, the feasibility of PARM-derived heterogeneity parameters is not restricted to 2D ultrasound imaging.

While the tumor model utilized in this study adequately demonstrates the feasibility of PARM in monitoring therapeutic progress, it does not fully replicate the complex vascular microenvironment of orthotopic brain implants and has limited metastatic potential 68. Future in-depth research will necessitate the use of more intricate orthotopic or spontaneous tumor models.

It's essential to clarify that our research focuses on the validation of the PARM technique as a versatile tool for characterizing microvascular changes, and we believe its broader applicability beyond the specific drug-tumor model combination used in this investigation.

## Conclusions

The findings of this proof-of-concept study indicate that the proposed PARM strategy provides a reliable and timely measurement of tumor vascular heterogeneity at the microvascular level. We anticipate that PARM could potentially play a valuable role in dynamic monitoring and early-stage evaluation of cancer therapy, shedding new insight on personally tailored cancer management.

## Supplementary Material

Supplementary figures, methods, video.

## Figures and Tables

**Table 1:** Spearman correlation coefficients (r) of histological vessel maturity index and PARM-derived heterogeneity, functional, and structural parameters (n=9).

Structural parameters				Functional parameters			Heterogeneity parameters	
Metric		r		Metric	r		Metric	r
VT	-0.70			MS	0.45		PC	-0.57
VNP	-0.32			SE	-0.37		PD	-0.77
VD	-0.42			OV	-0.52		PV	-0.67
								

PARM, pattern recognition of microcirculation; VT, vessel tortuosity; VNP, vessel number per pixel; VD, vessel density; MS, mean speed; SE, speed entropy; OV, orientation variance; PD, pattern distance; PC, pattern count; PV, pattern variance.

**Table 2:** Spearman correlation coefficients (r) of histological vessel maturity index and ULM-derived functional and structural parameters (n=9).

Structural parameters			Functional parameters	
Metric	r		Metric	r
VT	-0.03		MS	-0.20
VNP	-0.42		SE	-0.20
VD	-0.42		OV	0.14

ULM, ultrasound localization microscopy; VT, vessel tortuosity; VNP, vessel number per pixel; VD, vessel density; MS, mean speed; SE, speed entropy; OV, orientation variance.

**Table 3:** The correlation between the relative (to baseline) changes of microvasculature after the first treatment and the vessel maturity index and volume change measured at the end of treatment (n = 6).

		Vessel maturity index		Volume change	
		r	Slope	r	Slope
S	VT	-0.90	-0.30	0.86	0.06
	VN	-0.77	-1.15	0.94*	0.27
	VNP	-0.73	-1.34	0.87	0.31
	VD	-0.66	-0.95	0.71	0.22
	NN	-0.78	-1.71	0.92*	0.40
W	VT	-0.001	-0.0005	0.26	0.02
	VN	-0.18	-0.08	0.03	0.03
	VNP	0.11	0.08	0.09	-0.01
	VD	0.34	0.17	-0.31	-0.02
	NN	-0.18	-0.10	0.14	0.06

S, sentinel microvasculature; W, whole vasculature; VT, vessel tortuosity; VNP, vessel number per pixel; VD, vessel density; NN, node number; VN, vessel number. Bonferroni-corrected significant difference test was applied. Statistically significant correlations are denoted with asterisks (* = P < 0.05).

## Abbreviations

ULM: ultrasound localization microscopy
MB: microbubble
SRUS: super-resolution ultrasound imaging
CEUS: contrast-enhanced ultrasound
PARM: pattern recognition of microcirculation
TSF: trail spread function
VMI: vessel maturity index
