# Supplementary material for: Pattern recognition of microcirculation with super-resolution ultrasound imaging provides markers for early tumor response to anti-angiogenic therapy
Source: Theranostics. 2024 Jan 20;14(3):1312–24. doi: 10.7150/thno.89306 (PMC10845201; doi:10.7150/thno.89306)
Supplement: Supplementary file 1 — Supplementary figures, methods, video. [file thnov14p1312s1.zip › SupplementalContent_Cleanver_figures.pdf]

Supplemental Figures

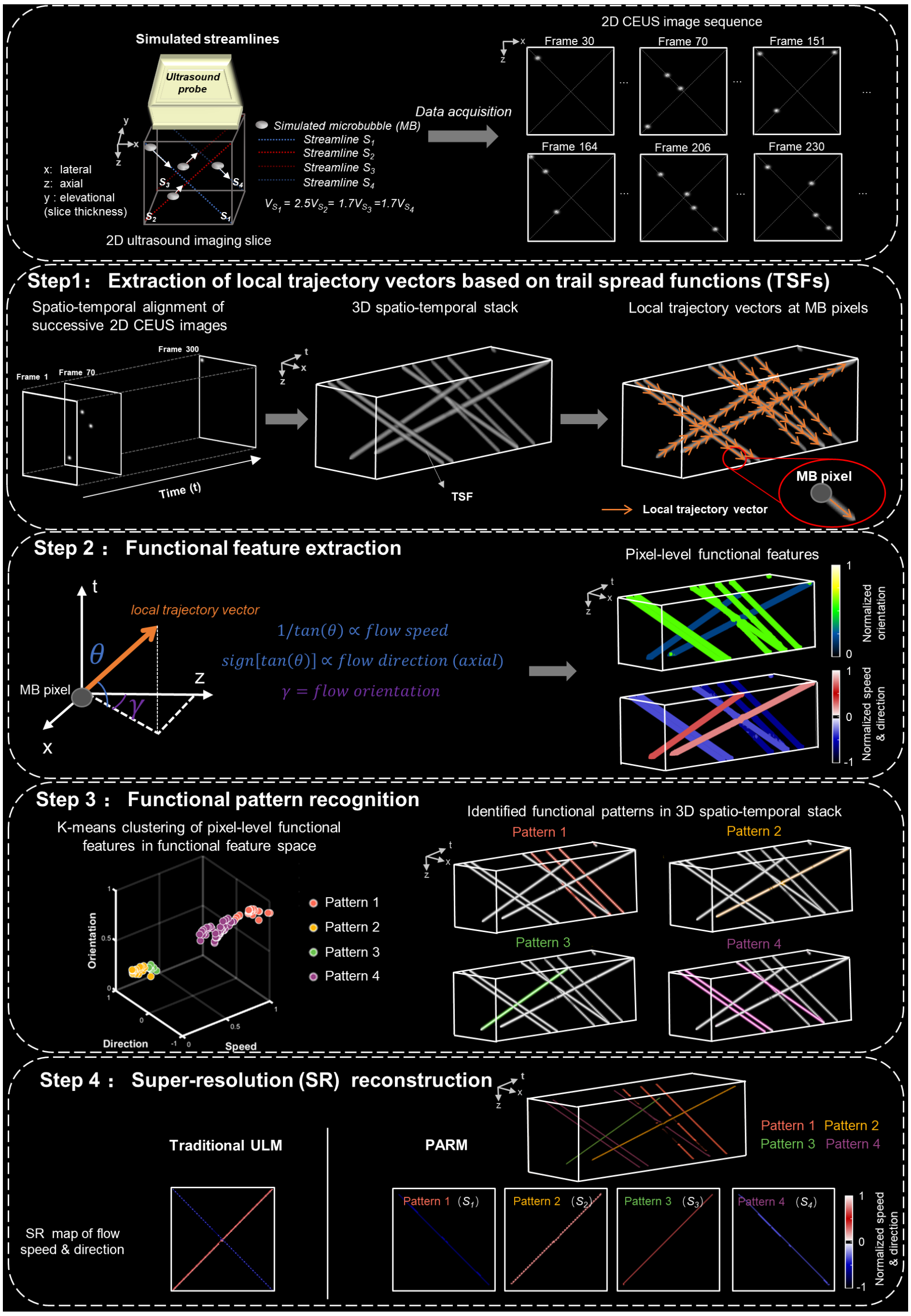

Figure S1. Schematic diagram of functional pattern recognition of microcirculation (PARM) process. Four streamlines with flowing microbubbles (MBs) were simulated. Compared with traditional ultrasound localization microscopy (ULM), vessels located very close to each other (S_1 and S_4 / S_2 and S_3) in the XZ plane can be effectively categorized, and flow characteristics can be more accurately quantified by PARM.

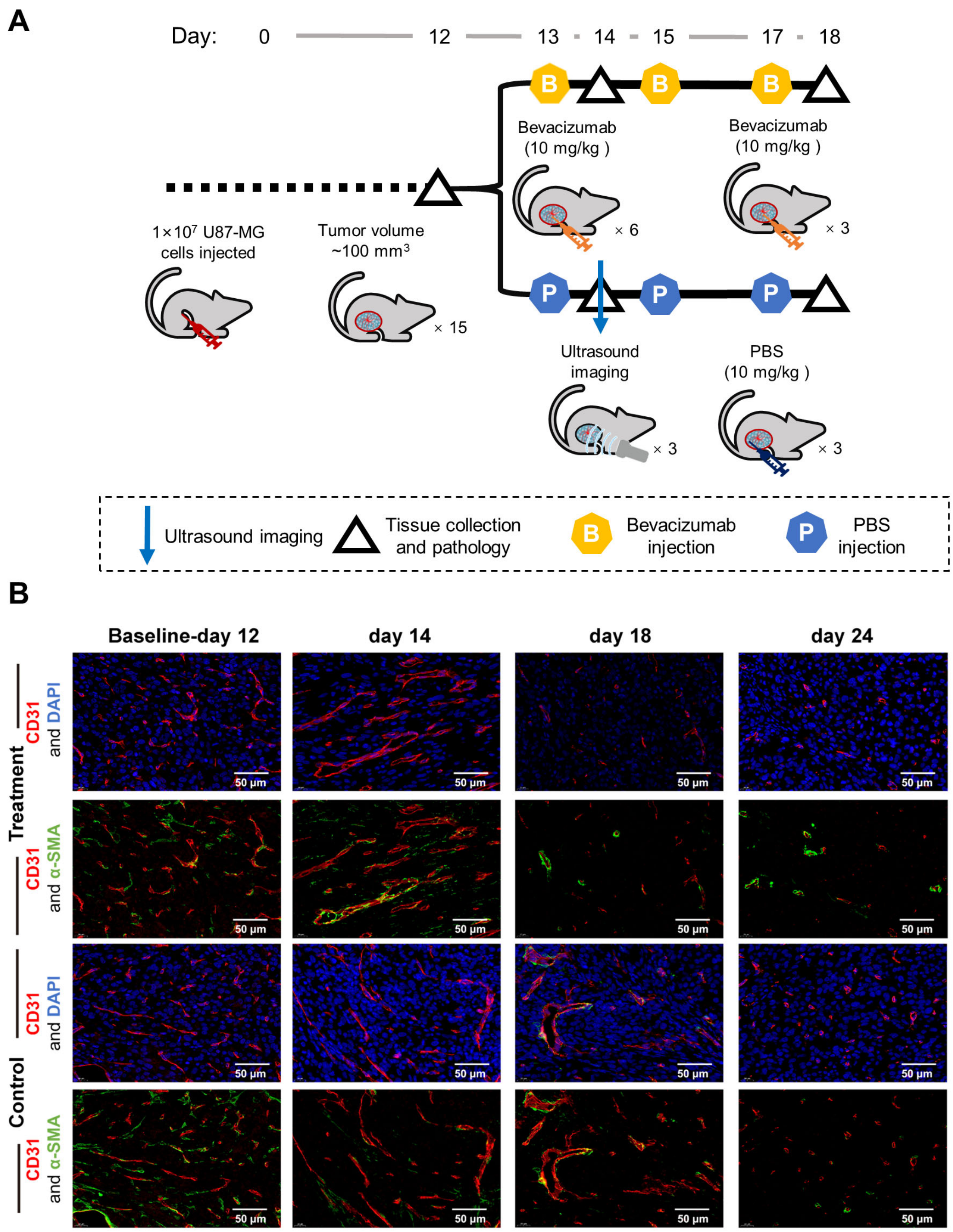

Figure S2. Study design and histological assessment of the longitudinal assessment of the anti-angiogenesis therapy group. (A) Procedure design of the longitudinal assessment of the anti-angiogenesis therapy group, including treatment, imaging, and immunohistochemical staining regimen in the treatment (bevacizumab injection) and the control (PBS injection) groups. (B) Representative partial images of tumor section stained by α-SMA (green) / CD31 (red), and DAPI (blue) /CD31 (red) in bevacizumab-treated and PBS-treated groups. Endothelial cells were detected by CD31 and perivascular cells by smooth muscle actin. Counterstaining of cell nuclei was performed with DAPI.

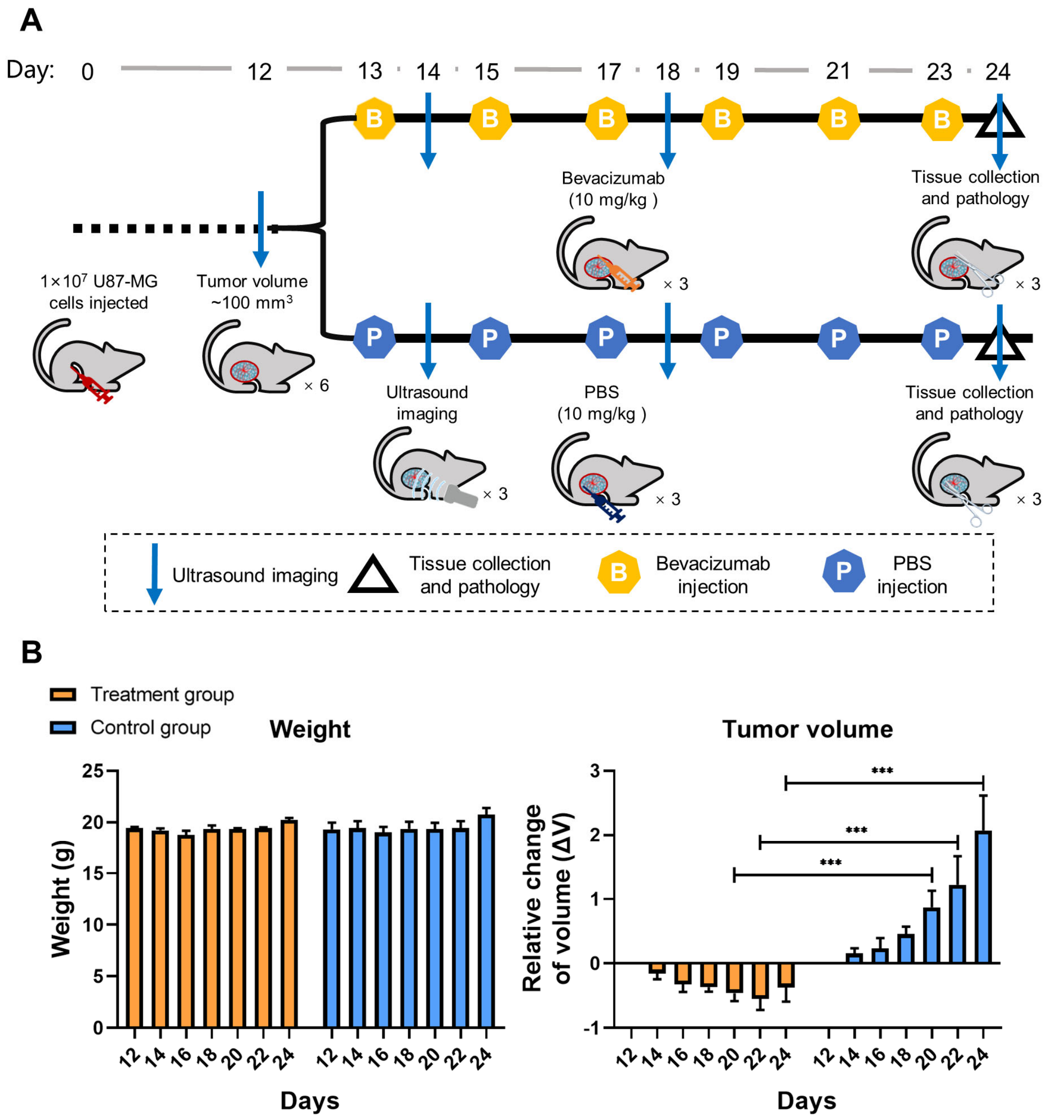

Figure S3. Study design and ultrasound imaging regimen of the longitudinal intraindividual ultrasound imaging group. (A) Procedure design of the longitudinal intraindividual ultrasound imaging group, including treatment, imaging, and immunohistochemical staining regimen in the treatment (bevacizumab injection) and the control (PBS injection) groups. (B) Monitoring tumor growth and body weight of tumor-bearing mice during treatment (* = P < 0.05, ** = P < 0.01, *** = P < 0.001).

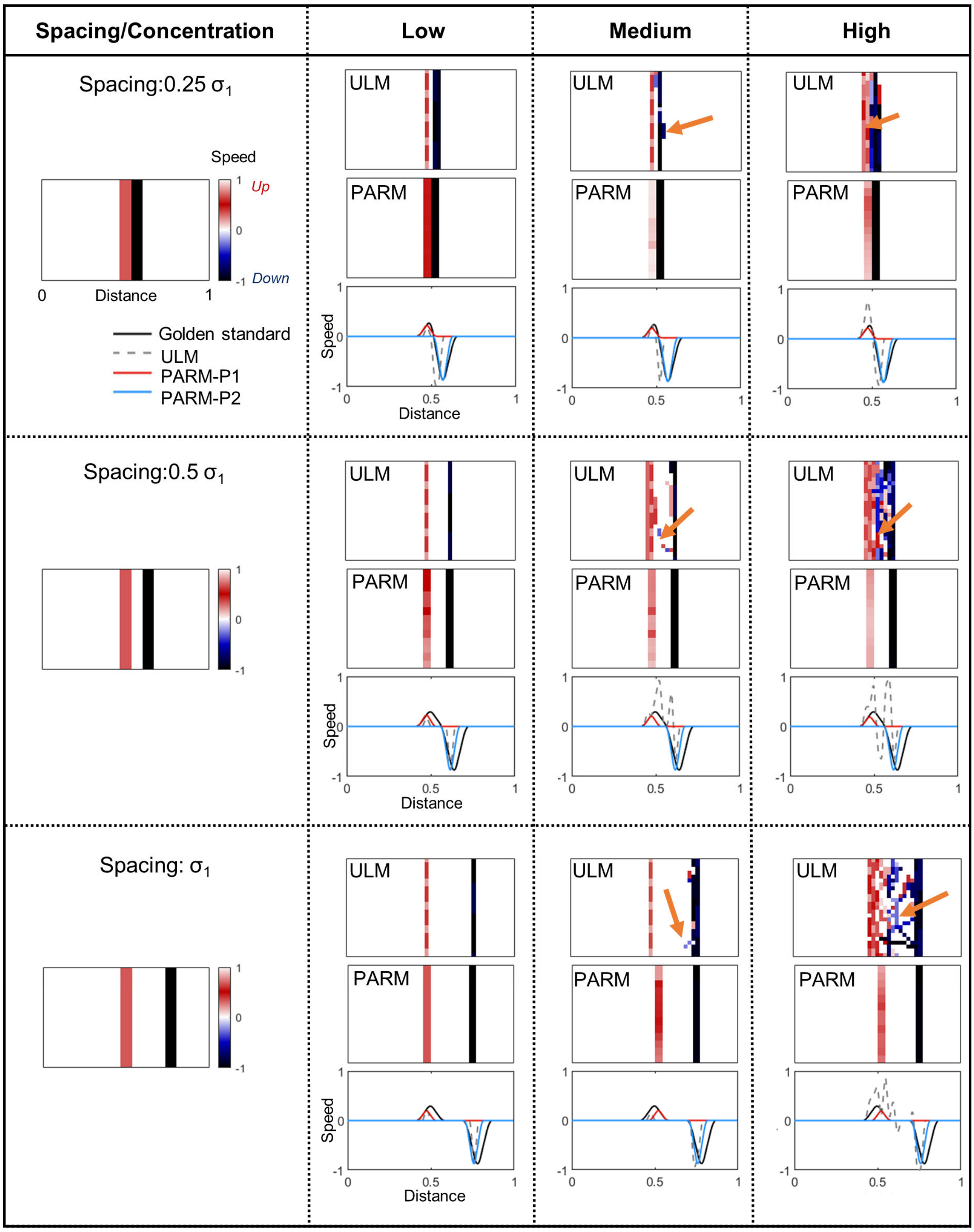

Figure S4. Simulated data to evaluate the performance of pattern recognition of microcirculation (PARM) in super-resolution reconstruction. The super-resolved velocity distributions of the ground truth, ultrasound localization microscopy (ULM), and PARM are compared, based on nine sets of contrast-enhanced ultrasound images with three MB concentrations and three streamline spacings. After PARM processing, super-resolved streamlines with different functional patterns are marked as PARM-P1 and PARM-P2. The standard deviation of simulated gaussian point spread function in the lateral direction is σ_1.

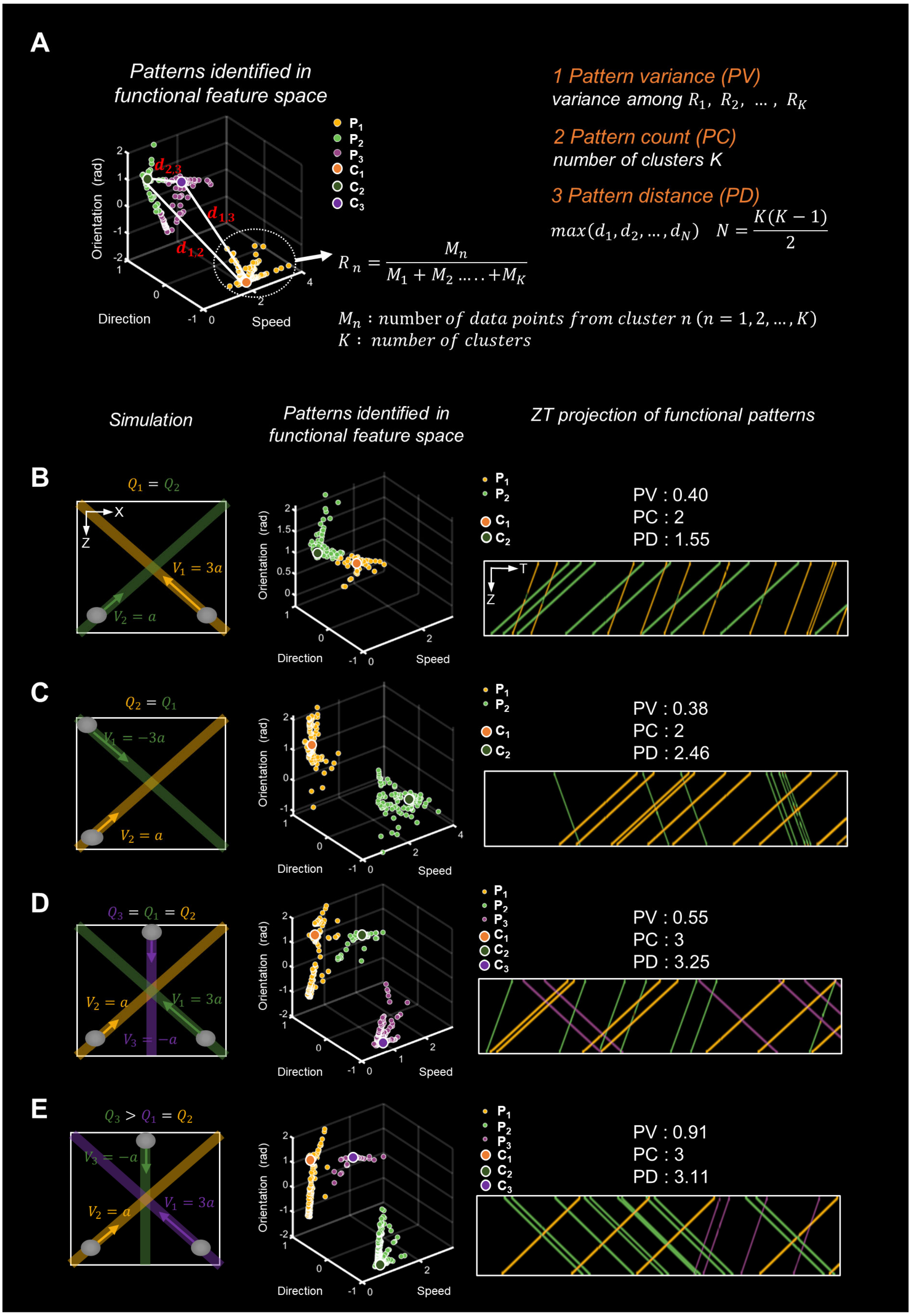

Figure S5. The relationships between the pattern recognition of microcirculation (PARM)-derived heterogeneity parameters and the functional characteristics of blood flow. (A) Assuming PARM identifies K (K = 3) functional patterns within the functional feature space, and designates these three patterns with distinct colors. The clustering centers for the identified functional patterns (P_1, P_2, P_3) are indicated by

white-edged markers: C_1 (center 1) in orange, C_2 (center 2) in green, and C_3 (center 3) in purple. Pixel-level functional features are color-coded accordingly. Heterogeneity parameters were computed to quantify pattern distributions within this space. (B-E) Simulated streamlines with different patterns were marked with distinct colors. Meanwhile, the patterns identified in the feature space, and ZT projection of simulated contrast-enhanced ultrasound image sequences were colored accordingly. Q represents the unit blood flow volume and was pre-set in each streamline. a represents the unit speed and was pre-set as well. PV, pattern variance; PD, pattern distance; PC, pattern count.

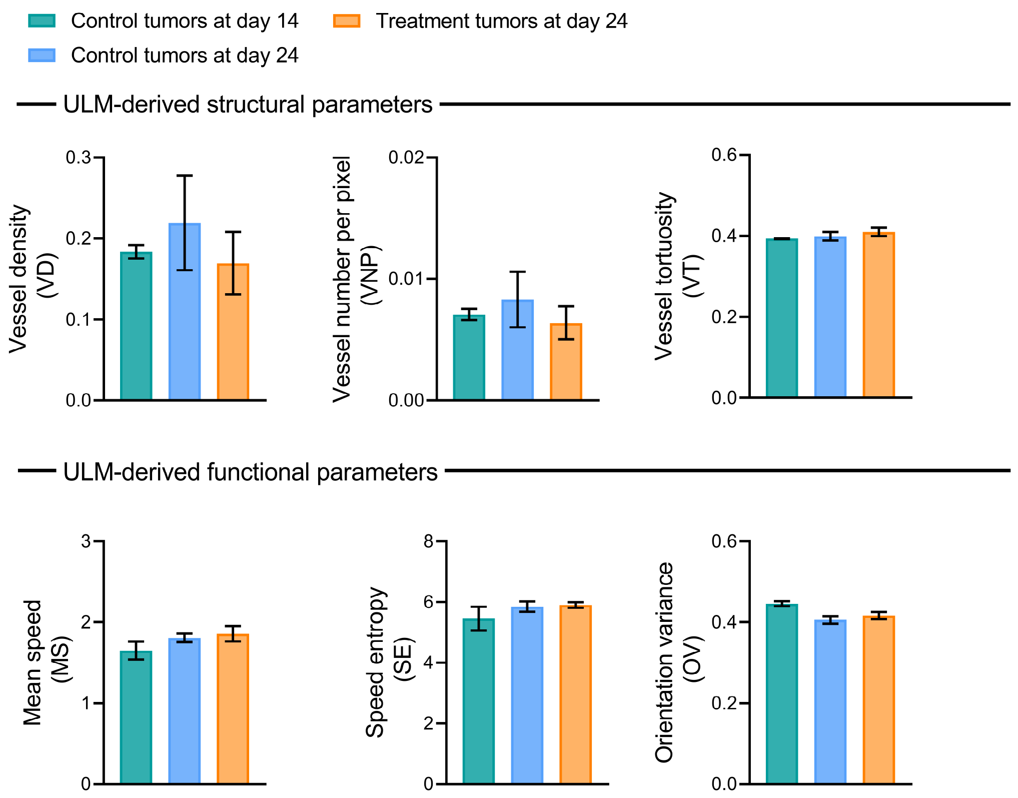

Figure S6. Ultrasound localization microscopy (ULM)-derived structural and functional parameters of 9 tumors with distinct physiological states. The functional and structural characteristics of 3 PBS-treated tumors at day 14 (green), 3 PBS-treated tumors at day 24 (blue), and 3 bevacizumab-treated tumors at day 24 (orange) are evaluated through ULM, and are compared with vessel density (VD-H) and vessel maturity index (VMI-H) from gold-standard fluorescent histology quantifications in the same tumor region (Figure 3). For all bar graphs shown, data are expressed as mean ± s.e.m. (n = 3 per group).

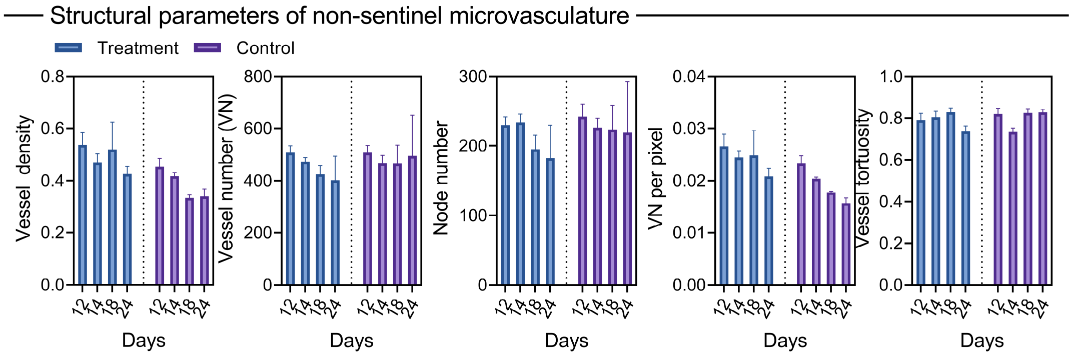

Figure S7. Morphological changes of non-sentinel microvasculature during anti-vascular treatment.

Supplementary Material

Characteristics of self-made microbubbles. The self-made microbubbles (MBs) were prepared for comparison with the proposed plasma microbubbles in [1]. The number-weighted diameter of OFP microbubbles was 800 nm. Compared with Sonovue, the contained gas of our self-made OFP microbubbles is identical and the size of OFP microbubbles is smaller. The same microbubble has been used in our previous work and has shown good enhancement of flow [2]. In terms of concentration, the microbubble concentration of Sonovue is generally in the range of 1×10^8 ml to 5×10^8 mL [3], which is an order of magnitude lower than the concentration we used.

Supplementary Methods

Simulation. MBs were simulated as Gaussian point spread function (PSF) with a standard deviation of σ_1 (lateral direction) and σ_2 (axial direction, $\sigma_2 = 0.75\sigma_1$). And a total of 1, 000 frames were generated for each simulated data set.

In this study, two types of simulated contrast-enhanced ultrasound (CEUS) images were generated.

Firstly, to investigate the capability of PARM in separating adjacent vessels over a wide range of imaging conditions (Figure S4), nine sets of CEUS data corresponding to three MB concentrations and three spacings were simulated. Specifically, different numbers of MBs flowing along the streamlines were chosen to simulate divergent concentrations. To produce an overlap between the MBs, the spacings between the two streamlines were empirically selected as $0.25\sigma_1$, $0.5\sigma_1$, and σ_1 respectively. These values represent various vessel densities relevant for small vessels like those imaged in the in vivo experiments. The flow velocity in the left vessel was $0.25\sigma_1$/frame, while the flow velocity in the right vessel was $-0.75\sigma_1$/frame.

Secondly, to visualize the relationship between PARM-derived heterogeneity parameters and blood flow characteristics, CEUS datasets corresponding to four different hemodynamic scenarios were simulated, shown in Figure S5.

PARM Analysis. The process of PARM analysis was illustrated in Figure S1. Firstly, 3D TSFs of MBs were constructed in a 3D spatio-temporal matrix I corresponding set of $D_x \times D_z \times D_t$ samples (D_x, D_z, and D_t were respectively the number of spatial samples along the X-direction, the number of spatial samples along the Z-direction and the number of 2D CEUS frames), formed by the spatio-temporal alignment of

successive 2D CEUS images. Assume that R pixels were higher than the previously set threshold (0.1, grayscale range 0~1), which were regarded as MB pixels P (x_r, z_r, t_r) (r = 1, 2, 3, …, R). Then, the structure tensor S (x,y,z) at every MB pixel (x, y, z) was calculated by first smoothing I, using a smoothing kernel G_σ with a standard deviation of σ, followed by the calculation of gradients (typically using central-difference kernels in three orthogonal directions) to yield I_x, I_y, and I_z. The tensor was constructed from gradient products as [4, 5]. The eigenvector (principal gradient directions) with the largest eigenvalue holds information about the trajectory's orientation, and is defined as the local trajectory vector at pixel (x, y, z) [4, 5].

Secondly, instantaneous functional features of MBs at MB pixels were acquired (Figure S1). Every local trajectory vector corresponding to each MB pixel P (x_r, z_r, t_r) was defined in spherical coordinates by the polar angle and azimuthal angle, as θ_r (x_r, z_r, t_r) (r = 1, 2, 3, …, R) and γ_r (x_r, z_r, t_r) (r = 1, 2, 3, …, R). It can be inferred that the absolute value of 1/tan(θ) is proportional to the instantaneous flow speed, while the sign of 1/tan(θ) indicates the axial direction of MB flow (defined as direction, up flow is set to be 1; down flow is set to be -1). Azimuth (γ) represents the lateral MB flow direction (defined as orientation). Functional features are defined in (1-3).

$$V_r = |1/\tan(\theta_r)| \qquad r = 1, 2, 3, \ldots, R \tag{1}$$

$$D_r = \mathrm{sign}(1/\tan(\theta_r)) \qquad r = 1, 2, 3, \ldots, R \tag{2}$$

$$O_r = \gamma_r \qquad r = 1, 2, 3, \ldots, R \tag{3}$$

Thirdly, to identify functional patterns of blood flows, the above functional features quantified at MB pixels were paired as $\{(D_r, O_r, V_r) \mid r = 1, 2, 3, \ldots, R\}$, forming a high-dimensional functional feature space. Different functional patterns of blood flow were then identified by performing adaptive K-means clustering.

To ensure the robustness of the clustering results and PARM parameters, we have maintained consistency in data collection parameters, including imaging center frequency, frame rate, and microbubble injection. For each specified K value, we performed the K-means clustering 10 times, each time with a new set of randomly initialized points. Moreover, the optimal number of clusters K is determined using the Davies-Bouldin index [6]. Compared with other clustering evaluation metrics, Davies-Bouldin index is insensitive to noise and outliers, and can correctly identify clusters under the influence of unbalanced cluster densities. These makes it suitable for the efficient identification of the blood flow patterns within tumor showing uneven flow distributions [7].

Correspondingly, all MB pixels P (x_r, z_r, t_r) were divided into K groups, forming K sparser MB subsets exhibiting different flow characteristics, defined as M_i (i = 1, 2, 3, …, K)

Finally, each MB subset M_i was processed separately to obtain several super-resolved microvessel images. Dynamic functional maps of blood flow at super-

resolution can be obtained by combining instantaneous MB flow directions and speeds quantified at the pixel level and super-resolution vascular images.

During PARM analysis, to minimize the risk of MB distortion resulting from overlapping microbubbles (intersecting TSFs) before splitting the them into subsets, we used Hessian filter to suppress the signals at crossing positions in 3D spatio-temporal stack at first. Besides, local trajectory vectors with anomalous directions and the corresponding pixels were discarded. The remaining undesired signals can be further suppressed by filtering in the super-resolution reconstruction (as has been discussed in [8]).

ULM processing. ULM maps were reconstructed through the ThunderSTORM plugin as previously established [9-11]. This plugin relies on local maxima detection and weighted maximum likelihood estimation for Gaussian fitting, employing the Gaussian-fitted centroids as estimated localization centers. Prior to fitting, Gaussian filtering was applied by the plugin for noise reduction. The PSF model was configured as an integrated Gaussian, with a fitting radius of 3 pixels. The fitting algorithm employed was the maximum likelihood estimator, with an interpolation factor set at 3.

For microbubble tracking in Figure S4, a tracking algorithm based on the Kuhn-Munkres assignment method was chosen (simpletracker, Mathworks, https://github.com/tinevez/simpletracker). After tracking, we systematically excluded extremely short trajectories, only trajectories with continuous tracking for five frames or more were retained to enhance imaging quality. The velocity map was computed based on their average velocity.

Assessment of traditional vascular parameters. Based on the binarized super-resolved vessel images, vessel density (VD) was defined as the total number of foreground (vascular) pixels in the binarized image divided by the area of the manually selected tumor region. Vessel skeletons were extracted by refinement algorithm [12], then converted into a network graph described by nodes and edges [13], thus vessel number (VN) and node number (NN) parameters were obtained. Vessel number per pixel (VNP) was quantified as the number of vessels divided by the total number of pixels of the tumor region [14]. The average sum of the angles (SOAM) value of all vessels was taken as the vessel tortuosity (VT) value of the tumor [15]. Mean speed (MS) was taken as the average value of the flow speed [16]. Speed entropy (SE) was defined as the information entropy of the local flow direction. Orientation variance (OV) was obtained by calculating the variance of local flow orientation [17].

Vascular metrics for each imaging slice were averaged to represent the overall

vascular characteristics of the tumor.

Supplemental References

1. Dong F, Zhang J, Wang K, Liu Z, Guo J, Zhang J. Cold plasma gas loaded microbubbles as a novel ultrasound contrast agent. Nanoscale. 2019; 11: 1123-30.

2. Yin J, Zhang J, Zhu Y, Dong F, An J, Wang D, et al. Ultrasound microvasculature imaging with entropy-based radiality super-resolution (ERSR). Phys Med Biol. 2021; 66: 215012.

3. Schneider M. Characteristics of SonoVue (TM). Echocardiography-J CARD. 1999; 16: 743-6.

4. Nelson LJ, Smith RA, Mienczakowski M. Ply-orientation measurements in composites using structure-tensor analysis of volumetric ultrasonic data. Compos Part A Appl Sci Manuf. 2018; 104: 108-19.

5. Westin CF, Maier SE, Mamata H, Nabavi A, Jolesz FA, Kikinis R. Processing and visualization for diffusion tensor MRI. Med Image Anal. 2002; 6: 93-108.

6. Davies DL, Bouldin DW. Cluster separation measure. IEEE Trans Pattern Anal Mach Intell. 1979; 1: 224-7.

7. Liu Y, Li Z, Xiong H, Gao X, Wu J, Wu S. Understanding and enhancement of internal clustering validation measures. IEEE Trans Cybern. 2013; 43: 982-94.

8. Huang C, Lowerison MR, Trzasko JD, Manduca A, Bresler Y, Tang S, et al. Short acquisition time super-resolution ultrasound microvessel imaging via microbubble separation. Sci Rep. 2020; 10: 6007.

9. Bar-Zion A, Solomon O, Tremblay-Darveau C, Adam D, Eldar YC. SUSHI: sparsity-based ultrasound super-resolution hemodynamic imaging. IEEE Trans Ultrason Ferroelectr Freq Control. 2018; 65: 2365-80.

10. Bar-Zion A, Tremblay-Darveau C, Solomon O, Adam D, Eldar YC. Fast vascular ultrasound imaging with enhanced spatial resolution and background rejection. IEEE Trans Med Imaging. 2017; 36: 169-80.

11. Gustafsson N, Culley S, Ashdown G, Owen DM, Pereira PM, Henriques R. Fast live-cell conventional fluorophore nanoscopy with ImageJ through super-resolution radial fluctuations. Nat Commun. 2016; 7: 12471.

12. Lee TC, Kashyap RL, Chu CN. Building skeleton models via 3-D medial surface/axis thinning algorithms. CVGIP Graph Models Image Process. 1994; 56: 462-78.

13. Kollmannsberger P, Kerschnitzki M, Repp F, Wagermaier W, Weinkamer R, Fratzl P. The small world of osteocytes: connectomics of the lacuno-canalicular network in bone. New J Phys. 2017; 19: 073019.

14. Yang Z, Chen J, Yao J, Lin R, Meng J, Liu C, et al. Multi-parametric quantitative microvascular imaging with optical-resolution photoacoustic microscopy in vivo. Opt Express. 2014; 22: 1500-11.

15. Shelton SE, Lee YZ, Lee M, Cherin E, Foster FS, Aylward SR, et al. Quantification of microvascular tortuosity during tumor evolution using acoustic angiography. Ultrasound Med Biol 2015; 41: 1896-904.

16. Lowerison MR, Huang CW, Lucien F, Chen SG, Song PF. Ultrasound localization microscopy of renal tumor xenografts in chicken embryo is correlated to hypoxia. Sci Rep. 2020; 10: 2478

17. Opacic T, Dencks S, Theek B, Piepenbrock M, Ackermann D, Rix A, et al. Motion model ultrasound localization microscopy for preclinical and clinical multiparametric tumor characterization. Nat Commun. 2018; 9: 1527
